# Grey matter degeneration during multiple sclerosis is linked to activation of neuronal necroptosis by oxidized phosphatidylcholines

**DOI:** 10.21203/rs.3.rs-10050556/v1

**Published:** 2026-07-03

**Authors:** Ruoqi Yu, Jian Park, Qurat Ul Ain, Gaili Yan, Rachel A. Dignean, Stephanie Zandee, Wendy Klement, Sandra Larouche, Chao Zheng, Justin J. Botterill, Alexandre Prat, Dorian B. McGavern, Yifei Dong

**Affiliations:** 1 Department of Biochemistry, Microbiology & Immunology, College of Medicine, University of Saskatchewan, Saskatoon, Saskatchewan, Canada; 2 Department of Microbiology & Immunology, Faculty of Science, the Life Sciences Institute, University of British Columbia, Vancouver, British Columbia, Canada; 3 Neuroimmunology Unit, the Research Center of the Centre Hospitalier de l’Université de Montréal (CRCHUM), Department of Neuroscience, Faculty of Medicine, Université de Montréal, Montréal, Quebec, Canada; 4 Brain Health Imaging Centre and the Azrieli Centre for Neuro-Radiochemistry at the Centre for Addiction and Mental Health, Departments of Psychiatry, Chemistry, Pharmacology and Toxicology, University of Toronto, Toronto, Ontario, Canada; 5 Department of Anatomy, Physiology & Pharmacology, College of Medicine, University of Saskatchewan, Saskatoon, Saskatchewan, Canada; 6 Viral Immunology and Intravital Imaging Section, National Institute of Neurological Disorders and Stroke (NINDS), National Institute of Health (NIH), Bethesda, MD, USA.

## Abstract

Oxidized phosphatidylcholines (OxPCs) are biomarkers of oxidative stress found in grey matter (GM) lesions during multiple sclerosis (MS), yet their distinct role in GM neurodegeneration remains undefined. Here we report that stereotaxic OxPC deposition in the mouse spinal cord GM induces age dependent neuroinflammation and neurodegeneration. Microglia are the predominant macrophages responding to OxPC induced GM lesions and help to mitigate acute neurodegeneration. Neuronal necroptosis activation in mouse GM lesions and neuronal upregulation of OxPCs and necroptosis activation in MS GM lesions suggest OxPC induced necroptosis promote GM degeneration during MS. In support, necroptosis inhibition ameliorates OxPC induced GM neuron loss. Finally, iron(ii)-containing heme deposition in the GM induces both OxPC formation and neuronal necroptosis activation, suggesting an endogenous upstream mechanism for generating neurotoxic OxPCs. These results highlight a plausible link between heme deposition, lipid peroxidation, and neuronal loss, and that necroptosis inhibition could help prevent GM neurodegeneration during MS.

## Introduction

While multiple sclerosis (MS) is classically defined by neuroinflammation, demyelination, and axonal degeneration in the white matter (WM) of the central nervous system (CNS), widespread grey matter (GM) injury also occurs throughout different stages of this disease^1–5^. Indeed, focal demyelinating lesions and diffuse atrophy are found across different CNS GM regions during MS including the cerebral cortex, cerebellum, deep grey matter, and spinal cord^6–9^. Importantly, GM volume loss correlates with the accumulation of cognitive impairments and disability during MS, indicating that brain and spinal cord atrophy resulting from GM degeneration contributes to MS disease progression^5,10–14^. Despite these clinical observations, the cellular and molecular processes that underlie GM pathology during MS remain poorly understood, thus limiting the development of effective treatments to stop the neurodegeneration and disabilities that accumulate due to MS progression.

Oxidized phosphatidylcholines (OxPCs) are lipid peroxidation byproducts^15^ that accumulate and associate with damaged neurons, axons, and reactive glial cells found in CNS lesions during all stages of MS progression^16–26^. Their elevation in the cerebrospinal fluid during MS suggests that OxPCs either form or disseminate endogenously in the CNS whereas the generation of autoantibodies against OxPC derivatives in MS brains indicates a systemic immune response against endogenous OxPC accumulation^27,28^. In addition to being biomarkers of oxidative injury, OxPCs can hyperactivate immune cells^29^, disrupt plasma membrane integrity^30^, damage mitochondria^31^, and promote cell death by apoptosis^32,33^ or ferroptosis^34,35^. These findings, together with studies highlighting OxPCs as mediators of tissue injury in chronic non-CNS diseases^36,37^, suggest that OxPC accumulation during MS is detrimental to CNS integrity. Indeed, we previously demonstrated that OxPCs found in WM lesions during MS promote both acute and chronic pathology in the spinal cord WM of young and middle-aged mice^22–25^, indicating that OxPCs deposition contribute to WM injury during relapse-remitting and progressive MS. Given OxPCs also directly induce neuronal death^22^ and accumulate in areas with GM neurodegeneration during MS^16–20^, investigating how the GM responds to OxPC deposition will further illuminate OxPC associated GM pathology during MS. Furthermore, determining how neurons and microglia in the GM respond to OxPC exposure, whether OxPCs engage distinct cell death pathways in the GM distinctly from the WM, and what initiates aberrant OxPC accumulation in the GM, will help close knowledge gaps critical for developing effective therapeutics against GM neurodegeneration during progressive MS.

Building on our recently developed approach to study MS-relevant OxPC biology in the spinal cord WM of mice^22–25^, we aimed to investigate the neurotoxic capacity of OxPCs in the mouse spinal cord GM and to identify mechanisms important for mitigating OxPC induced GM neurodegeneration. Our results indicate that deposition of OxPCs in the GM rapidly promotes neuronal death, the severity of which was modulated by age and by microglia depletion. Notably, neurons in the GM have elevated levels of phosphorylated mixed lineage kinase domain-like protein (pMLKL) and phosphorylated receptor-interacting protein kinase 3 (pRIPK3) prior to OxPC induced death, and pharmacological inhibition of RIPK3 ameliorated OxPC induced neuron loss. These findings together with the observation that OxPCs and pMLKL accumulate in proximity with neurons found in GM lesions from progressive MS brains provide evidence that OxPC induced necroptosis may be an underlying mechanism of GM neurodegeneration in MS.

## Results

### OxPC deposition in the spinal cord GM induces acute neuroinflammation and neuronal destruction

**Figure 1:**
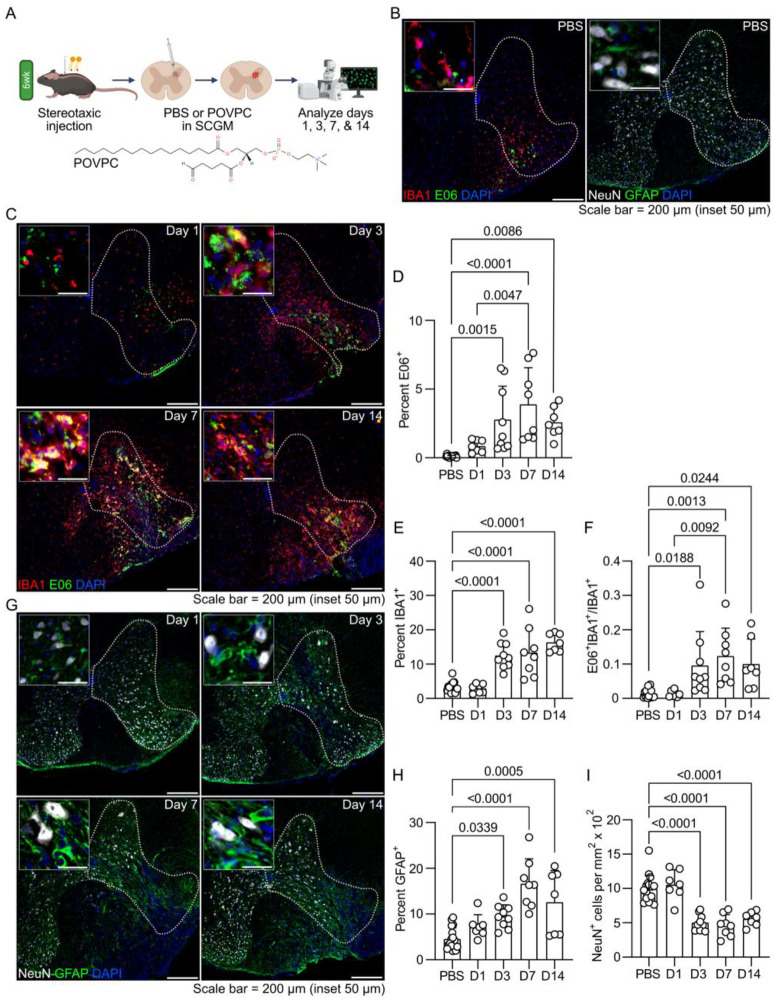
OxPC induces acute neuroinflammation and neurodegeneration in the spinal cord GM a) Schematic of experimental design analyzing OxPC lesions in the GM over 14 days. b) Representative immunofluorescence images of the spinal cord GM after PBS vehicle injection, labeled with DAPI (blue), IBA1 (red), and E06 (green) on the left, or with DAPI (blue), NeuN (grey), and GFAP (green) on the right. c) Representative images of the spina cord GM after 1, 3, 7 and 14 days post POVPC deposition, labeled with DAPI (blue), IBA1 (red), and E06 (green). d-f) Graphs comparing the amount of E06^+^ OxPCs (d) and IBA1^+^ cells (e) in the ipsilateral GM, as well as the proportion of IBA1^+^ cells that overlap with E06^+^ OxPCs (f) between PBS injected vehicle controls and different timepoints after POVPC deposition in the GM. g) Representative images of the spinal cord GM after 1, 3, 7 and 14 days post POVPC deposition, labeled with DAPI (blue), NeuN (grey), and GFAP (green). h-i) Graphs comparing the amount of GFAP^+^ astrocytes (h) and NeuN^+^ neuron density (i) in the ipsilateral GM between PBS injected controls and different timepoints after POVPC deposition in the GM. Data acquired from 2 separate experiments for each timepoint with n=17 mice for PBS injected controls, n=7 mice for day 1, n=9 mice for day 3, n=8 mice for day 7, and n=7 mice for day 14. Statistical significance reported as p-values, ordinary two-way ANOVA with Tukey’s multiple comparison test, comparing each group against each other. Data are represented as mean ± SD.

Previous lipidomic analyses identified 1-palmitoyl-2-(5′-oxo-valeroyl)-*sn*-glycero-3-phosphocholine (POVPC) as one of the most abundant OxPC species to accumulate in MS lesions^22,26^, and we found that its deposition in the CNS WM mediates oxidative injury with pathological features similar to MS^22–25^. To determine how OxPCs contribute to GM injury in MS, we performed a single stereotaxic injection of purified POVPC into the spinal cord GM of 6-week-old mice and compared tissue pathology after 1, 3, 7 and 14 days (Fig. 1a). Unlike an injection of phosphate-buffered saline (PBS) vehicle which caused minimal loss of GM tissue homeostasis (Fig. 1b), injection of POVPC induced significant neuroinflammation and neurodegeneration in the GM. Using the natural IgM antibody E06 which specifically binds to and detects OxPCs^36^, we found significant accumulation of E06^+^ OxPCs in the GM lesion only after 3, 7, and 14 days, but not at day 1 (Fig. 1c–d). This observation suggests that the POVPC which was injected initially had reacted completely with the tissue and that there may be endogenous formation of OxPCs in the GM lesion due to subsequent lipid peroxidation. This accumulation of E06^+^ OxPCs at days 3, 7, and 14 also coincided with an increased accumulation of ionized calcium binding adaptor molecule 1 (IBA1)^+^ microglia/macrophages and glial fibrillary acidic protein (GFAP)^+^ astrocytes in the GM lesion (Fig 1c, e, g-h). Notably, the overlap of IBA1^+^ immunoreactivity with E06^+^ immunoreactivity in the GM was significantly elevated on days 3, 7, and 14 (Fig. 1c, f), perhaps because microglia/macrophages have internalized newly formed OxPCs to minimize secondary tissue damage.

**Extended Figure 1:**
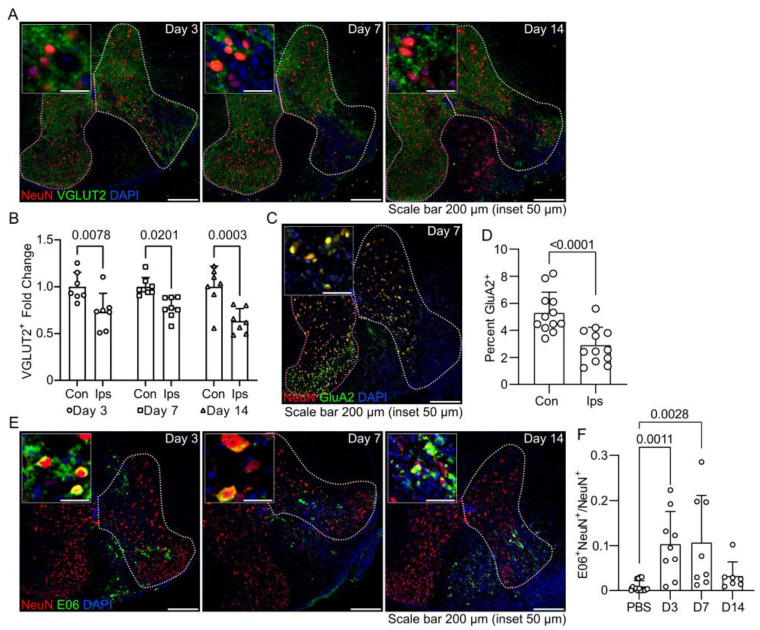
Additional evidence of OxPC induced GM neurodegeneration a) Representative images of spinal cord GM lesions 3, 7 and 14 days after POVPC injection, labelled with NeuN (red), VGLUT2 (green) and DAPI (blue). b) Graph comparing relative fold change in VGLUT2 expression between the contralateral GM and the ipsilateral GM lesion at days 3, 7, and 14. c) Representative image of spinal cord GM lesions 7 days after POVPC injection, labelled with NeuN (red), GluA2 (green) and DAPI (blue). d) Graph comparing percent GluA2 expression between the contralateral GM and the ipsilateral GM lesion on day 7. e) Representative images of spinal cord GM lesions 3, 7 and 14 days after POVPC injection, labelled with NeuN (red), E06 (green) and DAPI (blue). f) Graph comparing proportion of NeuN^+^ neurons overlapping with E06^+^ OxPCs in the ipsilateral GM in PBS injected mice or mice with lesion at days 3, 7, and 14. Data acquired from 2 separate experiments with n=7 mice for day 3, n=8 mice for day 7, and n=7 mice for day 14 in (b), or n=12 mice in (d), or n=12 for PBS, n=9 for day 3, n=8 for day 7, and n=7 for day 14 in (f). Statistical significance reported as p-values, paired two-tailed t-test comparing the contralateral with the ipsilateral GM in (b) and (d), or one-way ANOVA with Tukey’s multiple comparison test comparing different time points against each other in (f). Data are represented as mean ± SD.

In addition to neuroinflammation, there was a significant loss of neuronal nuclei antigen (NeuN)^+^ neurons in the GM 3 days after OxPC injection; however, there was no additional neuronal loss after 7 and 14 days (Fig. 1g, i). Consistent with this observation, the ipsilateral GM also had significant loss of vesicular glutamate transporter 2 (VGLUT2) and glutamate receptor 2 (GluA2) immunoreactivity compared to the unaffected contralateral GM in the spinal cords of OxPC injected mice (Extended Fig. 1a–d). Finally, NeuN^+^ neurons in the GM lesion had significantly greater overlap with E06^+^ OxPCs at days 3 and 7 (Extended Fig. 1e–f). Together, these findings indicate that OxPC induced acute lipid peroxidation and neurodegeneration in the GM.

### Microglia/macrophages responding to OxPC induced GM lesions express inflammatory signatures found in MS GM lesions

**Figure 2:**
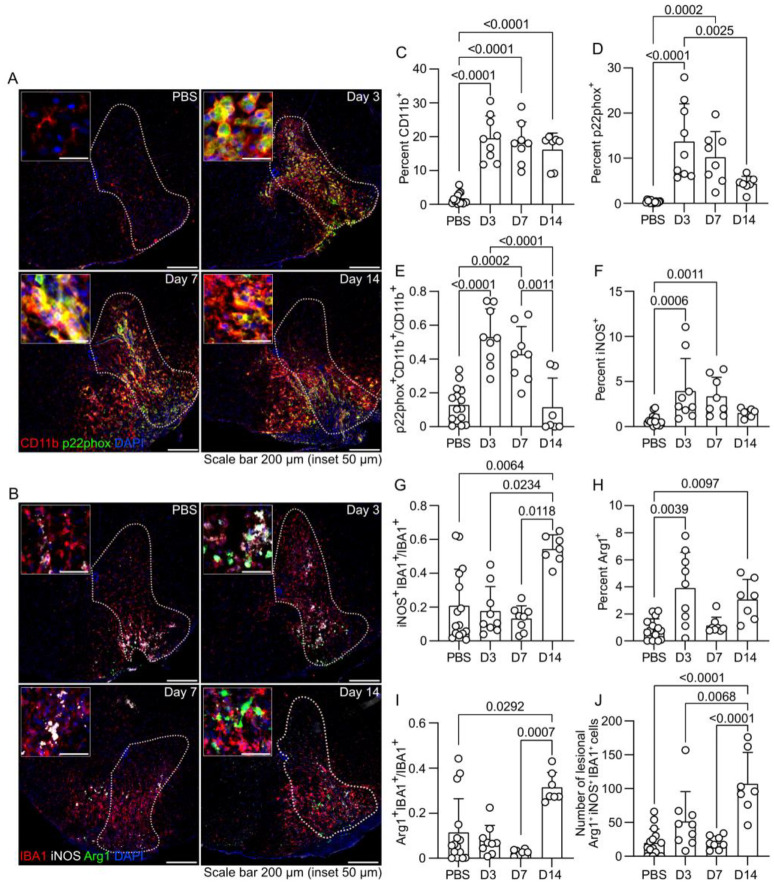
Characterizing the abundance and phenotypic changes in microglia/macrophages responding to GM oxidative injury a-b) Representative immunofluorescence images of the spinal cord GM injected with PBS, or days 3, 7, and 14 post POVPC injection labeled with DAPI (blue), CD11b (red), and p22phox (green) (a), or with DAPI (blue), IBA1 (red), iNOS (grey), and Arg1 (green) (b). c-j) Graphs comparing the percent of CD11b^+^ cells (c), percent p22phox expression (d), proportion of CD11b^+^ cells that overlapped with p22phox (e), percent iNOS expression (f), proportion of IBA1^+^ cells that overlapped with iNOS (g), percent Arg1 expression (h), proportion of IBA1^+^ cells that overlapped with Arg1 (i), and proportion of IBA1^+^ cells that overlapped with both iNOS and Arg1 (j) in the ipsilateral GM between PBS injected controls and different timepoints after POVPC deposition in the GM. Data was acquired from 2 separate experiments for each timepoint with n=15 mice for PBS control, n=9 mice for day 3, n=8 mice for day 7, and n=7 mice for day 14. Statistical significance reported as p-values, ordinary two-way ANOVA with Tukey’s multiple comparison test, comparing each group against each other. Data are represented as mean ± SD.

Since microglia/macrophages are key immune cells that react to GM injury during MS^3,19^, we further characterized phenotypic changes in microglia/macrophages responding to OxPC induced GM lesions. In deep GM brain lesions during MS, OxPC deposition correlates with the accumulation of NADPH oxidase subunit p22phox^+^ and induced nitric oxide synthase (iNOS)^+^ microglia/macrophages^19^. Similarly, there was significant elevation of p22phox and iNOS expression within OxPC induced GM lesions on days 3 and 7 (Fig. 2a–b, d, f). Interestingly, p22phox immunoreactivity in CD11b^+^ cells was increased on days 3 and 7 (Fig. 2g), whereas iNOS immunoreactivity in IBA1^+^ cells only increased on day 14 but not earlier (Fig. 2g). In contrast, levels of arginase-1 (Arg1), which is an enzyme associated with inflammatory suppression in macrophages^38^, was significantly elevated in GME lesions on days 3 and 14 but not on day 7 (Fig. 2b, h). However, the association of IBA1^+^ cells with Arg1^+^ immunoreactivity was only significantly increased on day 14 but not on day 3 (Fig. 2i), which suggested that the ontogeny of Arg1^+^ cells differed between these two time points. Interestingly, a population of Arg1^+^ iNOS^+^ IBA1^+^ microglia/macrophages were also present on day 14 but not on days 3 and 7 (Fig. 2j), which indicated that the phenotype and function of microglia/macrophages were dynamic in response to the local lesion microenvironment.

### Microglia mitigate neuronal loss in OxPC induced GM lesions

**Figure 3:**
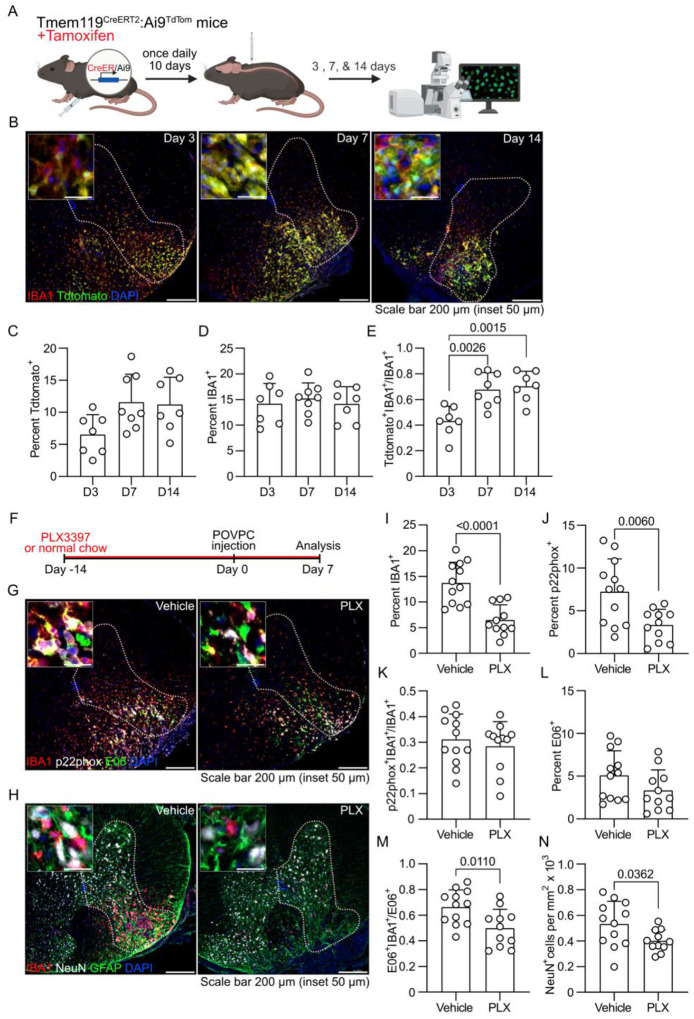
Microglia lineage tracing and depletion in OxPC induced GM lesions a) Schematic of experimental design for lineage tracing microglia in OxPC GM lesions from Tmem119 ^CreERT2^:Ai9^TdTom^ mice. b) Representative images of days 3, 7, and 14 GM lesions, labeled with DAPI (blue), IBA1 (red), and Tdtomato (green). c-e) Graphs comparing the percent Tdtomato^+^ cells (c), percent IBA1^+^ cells (d), and proportion of IBA1^+^ cells overlapping with Tdtomato (e) in the ipsilateral GM lesions at days 3, 7, and 14. Data acquired from at 2 separate experiments for each timepoint, with n=7 for day 3, n=8 for day 7, and n=7 for day 14. Statistical significance reported as p-values, two-tailed, one-way ANOVA with Tukey’s multiple comparison test, comparing each time points against each other. f) Schematic of experimental design for depleting microglia in OxPC induced GM lesions using the CSF1R inhibitor PLX3397. g-h) Representative images of day 7 OxPC induced GM lesions in mice with normal chow or chow containing PLX3397, labeled with DAPI (blue), IBA1 (red), p22phox (grey), and E06 (green) (g), or with IBA1 (red), NeuN (grey), and GFAP (green) (h). i-j) Graphs comparing the percent IBA1^+^ cells (i), percent p22phox^+^ cells (j), proportion of IBA1^+^ cells overlapping with p22phox (k), percent E06^+^ OxPCs (l), proportion of IBA1^+^ cells overlapping with E06^+^ OxPCs (m), and density of NeuN^+^ neurons (n) in the ipsilateral GM lesion epicenter between mice treated with normal chow and mice treated with chow containing PLX3397. Data acquired from 2 separate experiments with n=12 mice for normal chow and n=11 mice for chow with PLX3397. Statistical significance reported as p-values, two-tailed unpaired t-test, comparing normal chow with PLX3397. Data are represented as mean ± SD.

Compared to WM lesions which contain both reactive microglia and monocyte-derived macrophages, GM lesions during MS appear predominantly enriched with TMEM119 and P2RY12 expressing microglia^39^. How these microglia contribute to GM injury during MS remain unclear. To further delineate the dynamic contributions of microglia versus monocyte derived macrophages in the GM lesions, we stereotaxically injected POVPC into tamoxifen treated Tmem119^CreERT2^:Ai9^TdTom^ mice and lineage traced microglia/macrophages after 3, 7 and 14 days (Fig. 3a). In these mice, lesional microglia were identified as IBA1^+^ Tdtomato^+^ cells whereas monocytes/monocyte derived macrophages were identified as IBA1^+^ Tdtomato^−^ cells. While there were no significant changes in the amount of Tdtomato and IBA1 immunoreactivity across these timepoints (Fig. 3b–d), the relative amount of IBA1^+^ Tdtomato^+^ significantly increased on days 7 and 14 compared to day 3 (Fig. 3e). These observations suggest that while monocyte-derived cells respond acutely to GM pathology, microglia became more predominant within GM lesions over time.

**Extended Figure 2:**
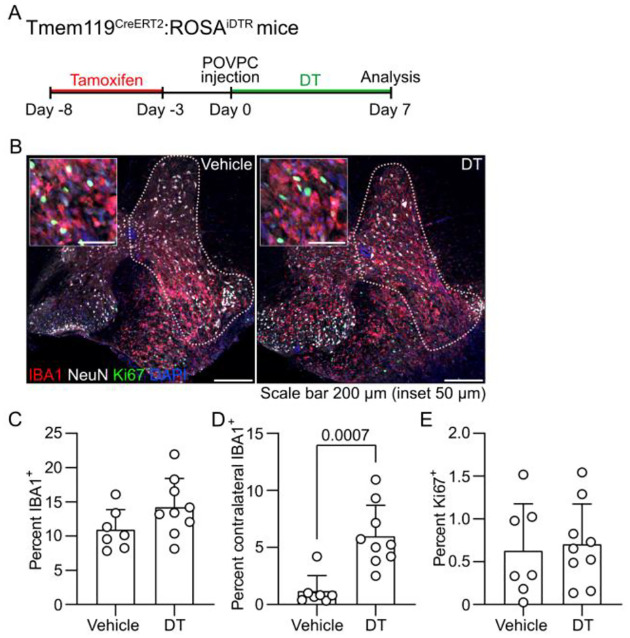
Diphtheria toxin treatment did not ablate microglia in Tmem119^CreERT2^:ROSA^iDTR^ mice. a) Schematic of experimental design for depleting microglia in OxPC induced GM lesions by injection of diphtheria toxin in tamoxifen treated Tmem119^CreERT2^:ROSA^iDTR^ mice. b) Representative images of day 7 GM lesions from vehicle and diphtheria toxin (DT) treated Tmem119^CreERT2^:ROSA^iDTR^ mice, labeled with IBA1 (red), NeuN (grey), Ki67 (green), and DAPI (blue). c-e) Graphs comparing the percent IBA1^+^ cells in the ipsilateral GM lesion (c), percent IBA1^+^ cells in the contralateral GM (d), and percent Ki67^+^ immunoreactivity in the ipsilateral GM lesion (e). Data acquired from 2 separate experiments with n=7 mice for vehicle treatment and n=9 mice for DT treatment. Statistical significance reported as p-values and unpaired two tailed t-test, comparing vehicle treatment with DT treatment. Data are represented as mean ± SD.

Since microglia are important for OxPC clearance in WM lesions^22,23,25^ and IBA1^+^ cells in OxPC induced GM lesions also overlapped with E06^+^ immunoreactivity (Fig. 1f), the microglia response is likely critical for clearing lesional OxPCs and for mitigating GM neurodegeneration. We first attempted to test this hypothesis by injecting vehicle or diphtheria toxin in tamoxifen treated Tmem119^CreERT2^:ROSA^iDTR^ mice for 7 days following POVPC injection in the spinal cord GM (Extended Fig. 2a). However, diphtheria toxin treatment did not induce a loss of IBA1^+^ cells in the ipsilateral GM lesion and even increased the amount of IBA1^+^ cells in the contralateral GM (Extended Fig. 2b–d), though this was independent of proliferation quantified by Ki67 (Extended Fig. 2e). Alternatively, we tested the effect microglia depletion by giving mice normal chow or chow containing the colony-stimulating factor 1 receptor (CSF1R) inhibitor PLX3397^40^ for 14 days prior to POVPC GM injection and for 7 days thereafter (Fig. 3f). At 7 days post lesion induction, PLX3397 treated mice had approximately 50% reduction in IBA1^+^ cells in the GM lesion compared to vehicle treated controls (Fig. 3g–i), indicating at least a partial depletion of microglial cells. Likewise, there was a significant reduction in the levels of lesional p22phox^+^ but no change in the amount of IBA1^+^ cells that it overlapped with (Fig. 3j–k). While total E06^+^ OxPC accumulation in GM lesions was unaffected by PLX3397 treatment (Fig. 3l), the proportion of IBA1^+^ that overlapped with E06^+^ immunoreactivity was significantly reduced after microglia depletion (Fig. 3m), potentially because microglia were involved in sequestering OxPC from the GM lesion microenvironment. More importantly, the density of NeuN^+^ neurons in the GM lesions were significantly reduced in PLX3397 treated mice compared to control mice, indicating that microglia loss exacerbated neurodegeneration (Fig. 3n).

### Aging reduces GM resilience against oxidative injury

**Figure 4:**
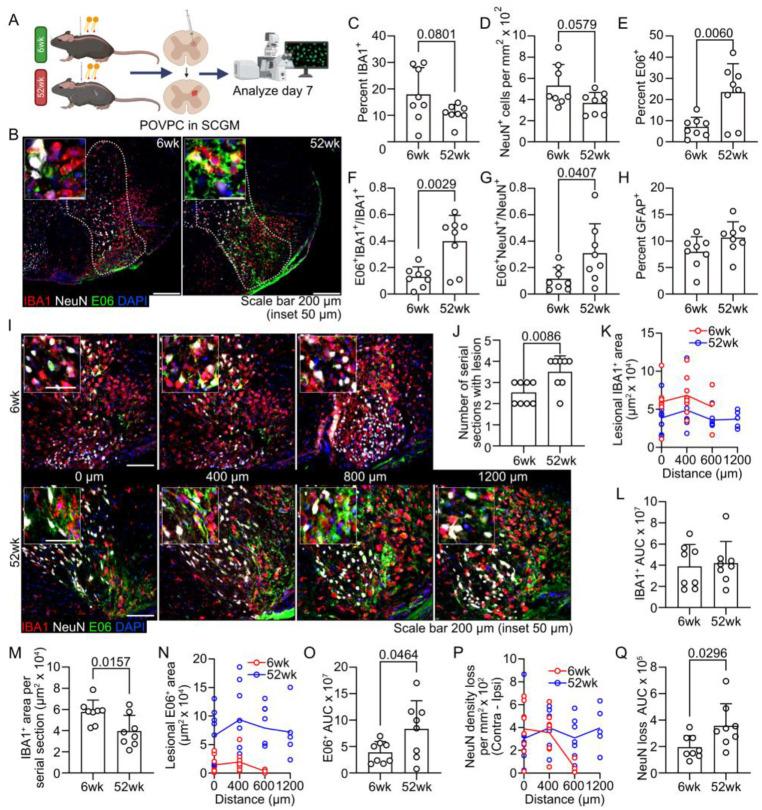
Aging dysregulates the microglia/macrophage response but exacerbates lipid peroxidation and neuronal loss in OxPC induced GM lesions a) Schematic of experimental design for comparing OxPC GM lesions between 6wk and 52wk old mice. b) Representative images of day 7 OxPC induced GM lesions in 6wk old and 52wk old mice, labeled with DAPI (blue), IBA1 (red), NeuN (grey), and E06 (green). c-h) Graphs comparing percent IBA1^+^ microglia/macrophages (c), NeuN^+^ neurons (d), E06^+^ OxPCs (e), proportion of IBA1^+^ cells overlapping with E06^+^ OxPCs (f), proportion of NeuN^+^ neurons overlapping with E06^+^ OxPCs (g), and percent GFAP^+^ astrocytes (h) in the ipsilateral GM lesion epicenter between 6wk and 52wk mice. i) Representative serial sections of the entire OxPC induced GM lesions in 6wk old and 52wk old mice, labeled with DAPI (blue), IBA1 (red), NeuN (grey), and E06 (green). j-q) Graphs comparing the number of GM-lesion containing serial sections (j), lesional IBA1^+^ area (k), total lesional IBA1^+^ area under the curve (AUC) (l), average IBA1^+^ area per serial section (m), lesional E06^+^ area (n), total lesional E06^+^ AUC (o), loss of NeuN^+^ neuron density (NeuN density in contralateral GM – NeuN density in ipsilateral GM) (p), and total loss of lesional NeuN^+^ neurons (q) across all serial sections in the ipsilateral GM between 6wk and 52wk mice. Data was acquired from 2 separate experiments with n=8 for 6wk mice and n=8 for 52wk mice. Statistical significance reported as p-values, two-tailed unpaired t-test, comparing 6wk lesions with 52wk lesions. Data are represented as mean ± SD.

Aging is linked to accelerated brain GM volume loss^41^, dementia^42^, and MS progression^43^, but observations on how aging directly affects oxidative inflammation and neurodegeneration in the GM during MS remains limited. To close this gap of knowledge, we injected POVPC into the spinal cord GM of young (6wk old) and middle-aged (52wk old) mice and analyzed OxPC induced GM pathology after 7 days (Fig. 4a). When comparing the epicenter of the GM lesion, 52wk old mice had a trend of reduced IBA1^+^ microglia/macrophages and NeuN density (Fig. 4b–d), as well as significantly increased levels of E06^+^ OxPC (Fig. 4b, e) and E06 immunoreactivity that overlapped with IBA1^+^ cells (Fig. 4f) and NeuN^+^ neurons (Fig. 4g). No change was observed in GFAP^+^ astrocytes (Fig. 4h).

More importantly, serial section analysis revealed that 52wk old mice had significantly more serial sections with GM pathology over the length of the spinal cord compared to 6wk old mice (Fig. 4i, j), which suggested that aging increased the GM lesion volume. Interestingly, while lesion-associated IBA1^+^ microglia/macrophages were detected in greater number of serial sections in 52wk old mice compared to 6wk old (Fig. 4k), the total estimated amount of IBA1^+^ cells that accumulated over the entire GM lesion was similar between 52wk old and 6wk old mice (Fig. 4l), and this is likely because aging lesions had significantly reduced accumulation of IBA1^+^ cells per serial section compared to young lesions (Fig. 4m). Furthermore, serial section analysis found that 52wk mice had significantly greater accumulation of E06^+^ OxPC (Fig. 4n–o) but significantly lower NeuN^+^ neuron density (Fig. 4p–q) compared to 6wk mice over the GM lesion volume. Collectively, these results indicate that the aging altered the behavior of microglia/macrophage as well as exacerbated lipid peroxidation and neurodegeneration during OxPC induced oxidative injury in the GM.

### OxPC induces necroptosis activation in neurons from the GM

**Extended Figure 3:**
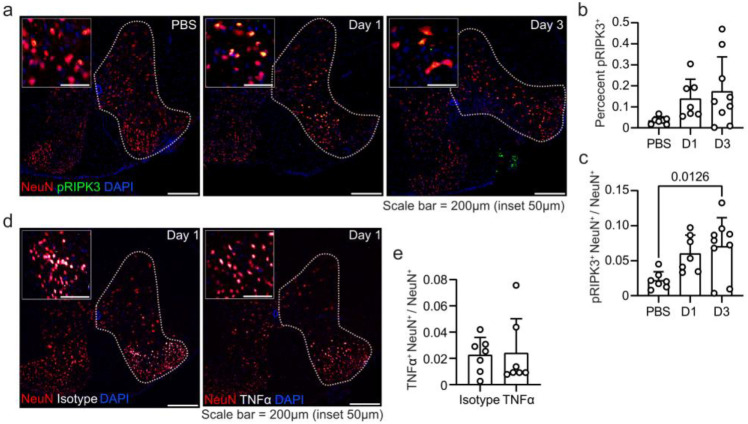
pRIPK3 and TNFα expression in OxPC induced GM lesions a) Representative images of spinal cord GM lesions 1 and 3 days after POVPC injection, labelled with NeuN (red), pRIPK3 (green) and DAPI (blue). b-c) Graph comparing percent pRIPK3 expression (b) and proportion of NeuN^+^ cells overlapping with pRIPK3 (c) in the ipsilateral GM in PBS injected mice or mice with lesions at days 1 and 3. d) Representative images of spinal cord GM lesions 1 day after POVPC injection, labelled with NeuN (red), isotype control antibody (grey) and DAPI (blue) on the left, or NeuN (red), TNFα (grey) and DAPI (blue) on the right. b-c) Graph comparing proportion of NeuN^+^ neurons overlapping with TNFα in the lesion one day after POVPC injection. Data acquired from at 2 separate experiments with n=7 mice for PBS, n=7 mice for day 1, and n=9 mice for day 7 in (b) and (c), or n=7 mice for day 1 grey matter stained with isotype or TNFα in (e). Statistical significance reported as p-values, two-tailed one-way ANOVA with Tukey’s multiple comparison test comparing PBS treated controls and GM lesions at days 1 and 3 in (b) and (c), or paired two-tailed t-test comparing day 1 grey matter stained with isotype or TNFα in (e). Data are represented as mean ± SD.

**Figure 5:**
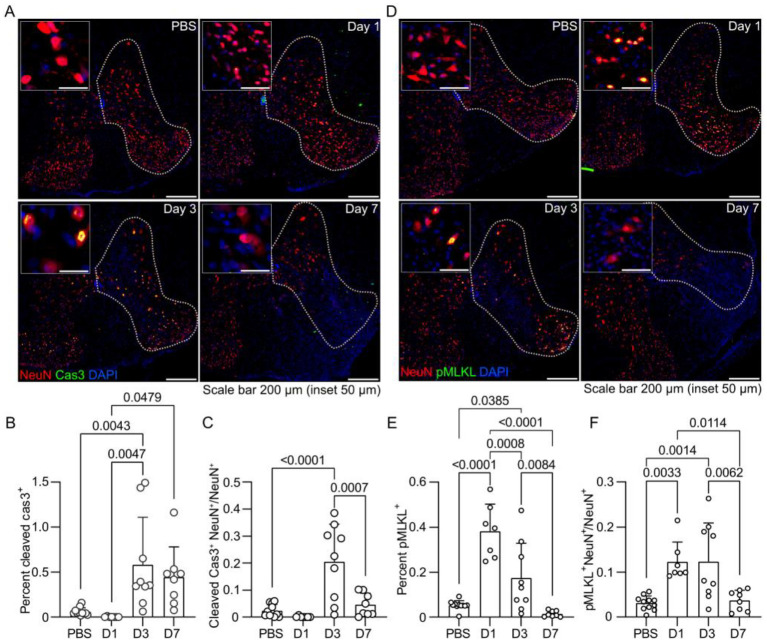
OxPC deposition in the GM induces rapid activation of neuronal necroptosis a) Representative immunofluorescence images of the spinal cord GM injected with PBS, or days 1, 3, and 7, post POVPC injection labeled with DAPI (blue), NeuN (red), and cleaved-caspase 3 (green). b-c) Graphs comparing percent cleaved-caspase 3 expression (b), and proportion of NeuN^+^ cells that overlapped with cleaved-caspase 3 (c) in the ipsilateral GM between PBS injected controls and different timepoints after POVPC deposition in the GM. d) Representative immunofluorescence images of the spinal cord GM injected with PBS, or days 1, 3, and 7 post POVPC injection, labeled with DAPI (blue), NeuN (red), and pMLKL (green). e-f) Graphs comparing percent pMLKL expression (e), and proportion of NeuN^+^ cells that overlapped with pMLKL (f) between PBS injected controls and different timepoints after POVPC deposition in the GM. Data was acquired from 2 separate experiments for each timepoint with n=11 mice for PBS control, n=7 mice for day 1, n=9 mice for day 3, and n=8 mice for day 7. Statistical significance reported as p-values, ordinary two-way ANOVA with Tukey’s multiple comparison test, comparing each group against each other. Data are represented as mean ± SD.

Understanding in the mechanisms that promote neuronal destruction in the GM during MS remains incomplete in part because commonly used animal models of MS do not adequately reflect the extent of oxidative injury found in humans^20^. Given that OxPCs accumulate in GM lesions during MS, we next explored potential mechanisms of OxPC mediated neurodegeneration in the mouse spinal cord GM. Quantification of NeuN^+^ cells in the ipsilateral GM following OxPC deposition showed that most of the neuronal death likely occurred between days 1 and 3 after lesion induction (Fig. 1i). As there was no additional loss of neurons by days 7 and 14, the cell death pathway that promoted OxPC induced acute neurotoxicity should also be the most active between days 1 and 3. OxPCs can induce apoptosis by activating cleaved-caspase 3^35,44^ and neurons treated with OxPCs *in vitro* have increased cleaved-caspase 3 activation^22^; thus, we first assessed the status of this pathway in GM lesions. While the total amount of cleaved-caspase 3 was elevated in the GM lesion at days 3 and 7, NeuN^+^ neurons only significantly associated with cleaved-caspase 3 immunoreactivity at day 3 but not at days 1 or 7 (Fig. 5a–c). However, since neurons from MS GM lesions upregulate necroptosis activators pMLKL and pRIPK3 but downregulate caspase-8 activity and cleaved-caspase 3 expression^45^, neuronal necroptosis may be a more predominant mechanism of GM neurodegeneration during MS compared to apoptosis. Given cleaved-caspase 3 was only elevated in neurons from GM lesions at day 3 (Fig. 5c), a time point after which significant neuronal loss had occurred (Fig. 1i), we next investigated necroptosis activation as an alternative pathway of OxPC induced GM neurodegeneration. Consistent with the hypothesis, pMLKL was significantly elevated in the GM lesion and within NeuN^+^ neurons at days 1 and 3 post OxPC deposition (Fig. 5d–f), and a similar result was observed in pRIPK3 expression (Extended Fig. 3a–c). Interestingly, since there was no substantial tumor necrosis factor-α (TNFα) immunoreactivity within GM lesions at day 1 (Extended Fig. 3d–e), necroptosis activation may be independent of TNFα activation at this specific timepoint.

### OxPC deposition associate with necroptosis activation in neurons from GM lesions found in progressive MS

**Figure 6:**
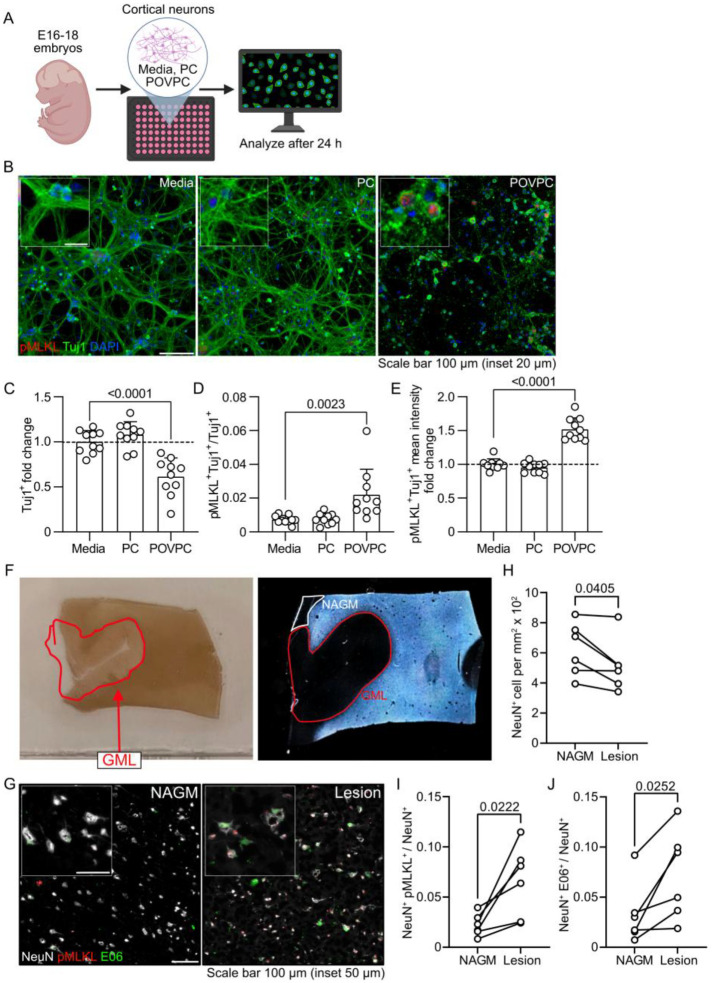
In vitro OxPC exposure upregulates pMLKL in neurons while elevation of OxPCs and pMLKL associate with neurons in progressive MS GM lesions a) Schematic of experimental design comparing mouse cortical neurons survival and pMLKL expression after 24 h treatment with media or 50 μM of non-oxidized PC, or 50 μM of POVPC. b) Representative immunofluorescence images of mouse cortical neurons after 24 h treatment with media alone, 50 μM PC, or 50 μM POVPC, labeled with DAPI (blue), Tuj1 (green), and pMLKL (red). c-e) Graphs comparing the relative survival of Tuj1^+^ neuron (c), the proportion of Tuj1^+^ neurons that overlapped with pMLKL (d), and relative change in pMLKL mean fluorescence intensity in Tuj1^+^ neurons (e) between media treated controls and PC or POVPC treatments. Data was acquired from 3 separate experiments with 4 technical replicates per condition per experiment. Statistical significance reported as p-values, ordinary one-way ANOVA with Dunnett’s multiple comparisons test, comparing each group against the control. Data are represented as mean ± SD. f) Representative bright field images of post-mortem brain tissue sections from individuals with progressive MS containing subpial GM lesion and NAGM. g) Representative immunofluorescence images of NAGM and the subpial GM lesion from the ROIs in (f), labeled with NeuN (grey), pMLKL (red) and E06 (green). h-j) Graphs comparing the density of NeuN^+^ neurons (h) and the proportion of NeuN^+^ cells that overlapped with pMLKL (h) or E06^+^ OxPC (i) between subpial GM lesions with the adjacent NAGM. Data was acquired from 6 individuals with secondary progressive MS. Statistical significance reported as p-values, two-tailed paired t-test, comparing GM lesions with the adjacent NAGM. Data represented as one dot per individual with progressive MS, where the NAGM and the GM lesion from each individual is connected by a straight line.

Although POVPC injection induced pMLKL and pRIPK3 in neurons from the spinal cord GM, it remained unclear whether this activation of necroptosis is caused by direct OxPC exposure or by secondary tissue damage. As most studies report that OxPCs promote cytotoxicity via apoptosis^32,33^ or ferroptosis^34,35^, whether OxPCs can directly promote necroptosis activation in neurons remains unknown. To test this possibility, we exposed primary mouse cortical neurons to non-oxidized dipalmitoylphosphatidylcholine (PC) or POVPC and analyzed neurotoxicity and necroptosis activation after 24 h (Fig. 6a). Compared to the media alone control, PC treatment did not affect neuronal viability quantified by tubulin β3 (Tuj1) integrity nor necroptosis activation quantified by pMLKL expression (Fig. 6b–d). In contrast, POVPC treated neurons caused significant reduction in Tuj1^+^ neurons and this was accompanied by significantly elevated overlap of pMLKL immunoreactivity with Tuj1^+^ neuronal debris. The mean intensity of pMLKL immunoreactivity that overlapped with Tuj1^+^ neurons was also significantly increased by POVPC treatment compared to PC and media alone controls (Fig. 6e). Finally, we assessed whether OxPCs accumulated with necroptosis activation in subpial GM lesions during MS by analyzing brain tissue sections isolated from rapid autopsy of 6 individuals with progressive MS^46,47^. Compared to the adjacent normal appearing GM (NAGM), the GM lesions had significantly reduced density of NeuN^+^ neurons, which was indicative of neurodegeneration (Fig. 6f–h). And consistent with previous findings showing pMLKL upregulation in neurons from MS cortical GM^45^, the levels of pMLKL immunoreactivity and E06^+^ OxPC accumulation that associated with NeuN^+^ neurons were significantly increased within GM lesions compared to the adjacent NAGM (Fig. 6i–j). Overall, these results suggest that OxPC accumulation in GM lesions associates with the activation of neuronal necroptosis during progressive MS.

### Pharmacological inhibition of RIPK3 partially ameliorates OxPC induced GM neurodegeneration

**Figure 7:**
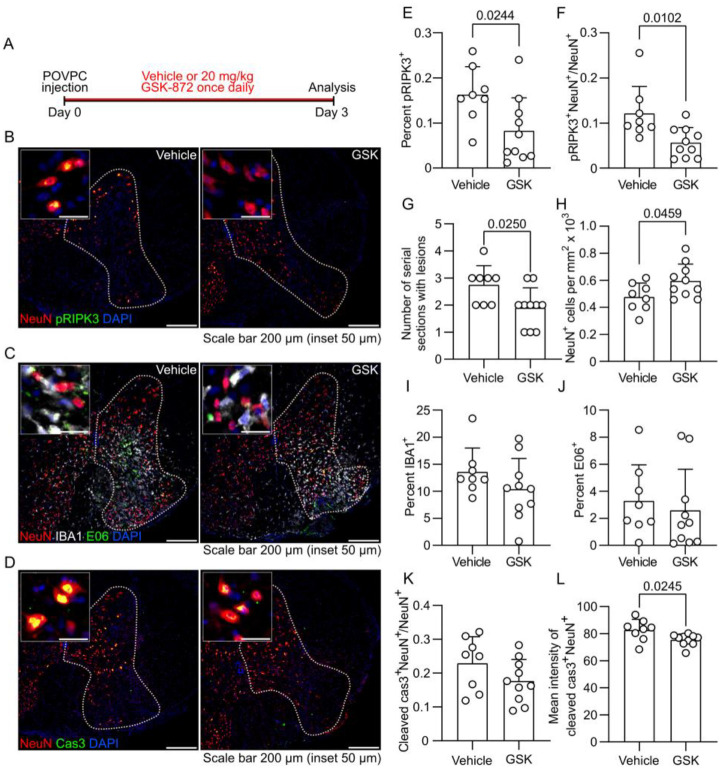
Inhibition of RIPK3 activation partly ameliorates neurodegeneration in OxPC induced GM lesions a) Schematic of experimental design using GSK-872 to inhibit RIPK3 activation in OxPC induced GM lesions. b-d) Representative images of day 3 OxPC induced GM lesions in mice with vehicle or with daily injection of 20 mg/kg GSK-872, labeled with DAPI (blue), NeuN (red) and pRIPK3 (green) (b), or with NeuN (red), IBA1 (grey), and E06 (green) (c), or with NeuN (red) and cleaved-caspase 3 (green) (d). e-m) Graphs comparing the percent pRIPK3^+^ in lesions (e), proportion of NeuN^+^ cells overlapping with pRIPK3 (f), total number of serial sections with lesion (g), density of NeuN^+^ neurons (h), percent IBA1^+^ microglia/macrophages (i), percent E06^+^ OxPCs (j), proportion of NeuN^+^ cells overlapping with cleaved-caspase 3 (k), and mean fluorescence intensity of cleaved-caspase 3 in NeuN^+^ neurons (l) in the ipsilateral GM lesion epicenter between mice treated with vehicle and mice treated GSK-872. Data acquired from 2 separate experiments with n=8 mice for vehicle and n=10 mice for GSK-872 treatment. Statistical significance reported as p-values, two-tailed unpaired t-test, comparing vehicle with GSK-872 treatment. Data are represented as mean ± SD.

Given that neuron-specific necroptosis activation in MS grey matter lesions^45^ occurs in proximity with E06^+^ OxPC deposition (Fig. 6g–i), and that OxPC deposition in the spinal cord GM induced neuronal loss (Fig. 1i) and activation of necroptosis mediators (Fig. 5e–f), we hypothesized that inhibiting this cell death pathway may be therapeutically effective against OxPC mediated neurodegeneration in the GM. Necroptosis activation is dependent on RIPK3 autophosphorylation^48^ and can be inhibited by GSK-872 which interferes with the activity of the RIPK3 kinase domain^49^. Thus, we treated mice with vehicle or 20 mg/kg of GSK-872 once per day following injection of POVPC in the spinal cord GM and analyzed the lesion after 3 days (Fig. 7a). Compared to control mice, GSK-872 treated mice had significantly reduced levels of pRIPK3 and reduced proportion of NeuN^+^ neurons overlapping with pRIPK3 immunoreactivity (Fig. 7b, e–f), which provided evidence that GSK-872 blocked RIPK3 activation in the GM lesion. More importantly, the number of serial sections containing GM lesions and the loss of NeuN^+^ cells in the GM lesion epicenter were significantly reduced by GSK-872 treatment (Fig. 7c, g–h). In contrast, there were no significant differences in the accumulation of IBA1^+^ microglia/macrophages and E06^+^ OxPCs between the two groups (Fig. 7i–j). Moreover, GSK-872 treatment did not change the number of NeuN^+^ neurons that overlapped with cleaved-caspase 3 expression (Fig. 7k) and only mildly reduced the mean intensity of cleaved-caspase 3^+^ immunoreactivity in these cells (Fig. 7l). Thus, inhibition of RIPK3 activity by GSK-872 treatment partially ameliorated OxPC induced GM neurodegeneration and may be an effective therapeutic approach to mitigate neuronal destruction in GM lesions during MS.

### Iron(ii)-containing heme deposition in the spinal cord GM induces endogenous lipid peroxidation and neuronal necroptosis

**Figure 8:**
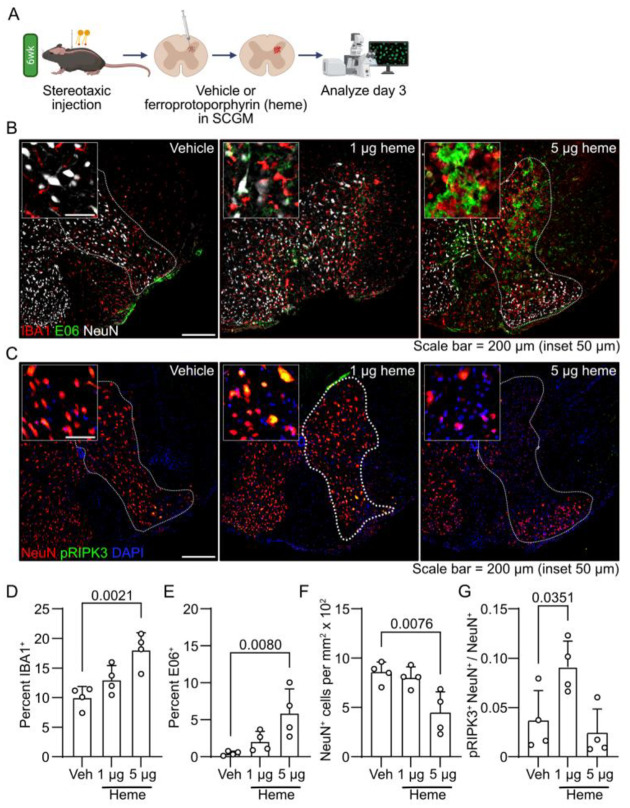
Deposition of iron(ii)-containing heme induces OxPC formation and neurodegeneration in the spinal cord GM a) Schematic of experimental design comparing spinal cord GM pathology in mice injected with vehicle or iron(ii)-containing heme (ferroprotoporphyrin). b-c) Representative immunofluorescence images of the spinal cord GM 3 days after vehicle, 1 μg, or 5 μg of iron(ii)-containing heme injection, labeled with IBA1 (red), E06 (green), and NeuN (grey) (b), or with DAPI (blue), NeuN (red) and pRIPK3 (green) (c). d-g) Graphs comparing percent of IBA1^+^ cells (d), percent E06^+^ OxPCs (e), density of NeuN^+^ neurons (f), and proportion of neurons that overlapped with pRIPK3 (g) in the ipsilateral spinal GM of mice injected with vehicle, 1 μg, or 5 μg of iron(ii)-containing heme. Data was acquired from 2 separate experiments for each timepoint with n=4 mice per treatment group. Statistical significance reported as p-values, ordinary one-way ANOVA with Dunnett’s multiple comparisons test, comparing each heme treated group against the vehicle control. Data are represented as mean ± SD.

Thus far, we found that OxPC deposition in the CNS GM promoted neuroinflammation and neurodegeneration potentially through necroptosis activation. Yet, the mechanisms that induce endogenous OxPC formation in the CNS especially within MS GM lesions remain unknown. Since iron accumulation in the CNS WM and GM strongly associates with MS pathology and disease progression^19,50,51^ and that iron(ii) can promote lipid peroxidation via the Fenton reaction^52^, neurotoxic OxPC formation in MS may be driven by iron(ii) accumulation. Furthermore, while the source of excess iron in MS remains unclear, there is emerging evidence suggesting that microhemorrhage^53^ and hemoglobin dysfunction may be contributing factors^54,55^. To test the hypothesis that aberrant hemoglobin accumulation in the CNS GM may promote OxPC formation and initiate neurodegeneration, we stereotaxically injected 1 μg or 5 μg of ferroprotoporphyrin, the iron(ii)-containing co-factor heme found in hemoglobin, into the mouse spinal cord GM and analyzed the tissue pathology after 3 days (Fig. 8a). Compared to vehicle injected mice, mice injected with 5 μg of heme had significantly greater accumulation of IBA1^+^ cells and E06^+^ OxPCs (Fig. 8b, d-e). A similar but milder response was observed in mice injected with 1 μg of heme. Interestingly, mice injected with 5 μg of heme had significantly reduced density of NeuN^+^ cells compared to vehicle injected mice (Fig. 8c, f) but pRIPK3 expression was only significantly associated with NeuN^+^ cells in mice injected with 1 μg of heme (Fig. 8c, g). This outcome is perhaps because necroptosis activation and neuronal destruction occurred more rapidly is mice treated with 5 μg of heme compared to mice treated with 1 μg of heme. Thus, the amount of endogenous OxPC generation as well as the rate of neuronal necroptosis activation and death are likely dependent on the availability of extracellular heme. Taken together, these observations indicate that deposition of iron(ii)-containing heme in the CNS GM promotes endogenous OxPC formation and neurodegeneration.

## Discussion

Despite strong evidence indicating that GM injury contributes to disease pathology and disability accumulation across all stages of MS^5–14,16–18,22,25^, the molecular mechanisms that underlie neuronal destruction in the GM during MS is not well understood. OxPCs are harmful byproducts of oxidative stress that accumulate in WM and GM lesions during MS^16–20,22,25^. Here, we report that OxPC deposition in the mouse spinal cord GM induced acute neuronal death and neuroinflammation, as well as potential endogenous lipid peroxidation. The intensity of neurodegeneration and lipid peroxidation in these GM lesions were significantly increased by aging and by microglia depletion. Mechanistically, OxPCs promoted rapid necroptosis activation in GM neurons in the mouse spinal cord, which mirrored OxPC accumulation and upregulation of the necroptosis mediator pMLKL in neurons from GM lesions during progressive MS. As pharmacological inhibition of RIPK3 partly ameliorated OxPC induced GM neurodegeneration, similar therapeutic approaches to block the activation of neuronal necroptosis may help to mitigate oxidative injury and rescue GM neuron loss during MS. Finally, while the cause of OxPC accumulation in MS remains unclear, iron(ii)-containing heme deposition in the GM induced significant OxPC formation, as well as neuronal necroptosis and death in a dose dependent manner. Collectively, these results highlight that OxPCs driven GM pathology is distinct from the WM and that iron(ii)-containing heme, OxPC deposition, and activation of neuronal necroptosis are potential mechanistic components that drive GM neurodegeneration in MS.

We previously reported that OxPC deposition in the CNS WM induces acute demyelination, neuroinflammation, as well as axonal degeneration^22,23^, and that the resulting focal lesion develops chronic pathology like chronic active lesions found in progressive MS^25^. In addition to understanding how OxPCs contribute to GM neurodegeneration during MS, comparing the extent and chronicity of OxPC induced GM pathology with WM pathology was a major aim of this study. Interestingly, the death of NeuN^+^ neurons in the GM mostly occurred between days 1 and 3 post POVPC deposition and there was no additional neuronal loss thereafter. Thus, unlike WM lesions^25^, OxPC induced lesions in the GM did not undergo additional self-sustaining neurodegeneration after the initial injury. Such pathological difference could be because compared to the GM, WM demyelination releases significant amounts of polyunsaturated fatty acids into the tissue environment^56^. This process in combination with inflammatory oxidative stress can lead to further lipid peroxidation, resulting in chronic OxPC accumulation which further perpetuates chronic WM injury. Since the GM has significantly less myelin content compared to the WM, the availability of lipids substrates to sustain lipid peroxidation and chronic neurodegeneration in GM lesions is likely more limited compared to the WM. Indeed, necroptosis activation peaked in neurons from GM lesions between days 1 and 3 and its level returned to baseline by day 7. Similarly, endogenous OxPC formation and neuroinflammation both peaked between days 3 and 7 but were in decline by day 14. Together, these observations indicate that oxidative injury in the GM evolved differently compared to the WM and therefore combinatorial therapeutic strategies may be necessary to simultaneously mitigate GM and WM neurodegeneration during MS.

There may be multiple mechanisms that potentiate OxPC accumulation in the GM. For instance, microglia and macrophages found in GM lesions during MS have high NADPH oxidase expression^19^, suggesting that they can produce ROS which in turn can oxidize PCs into OxPCs^37^. In contrast, recent evidence suggest mitochondria-derived ROS in monocyte-derived macrophages but not microglia are critical drivers of oxidative damage during the experimental autoimmune encephalomyelitis model of MS neuroinflammation^57^; this may be an NADPH oxidase independent pathway for generating ROS and OxPCs during MS. How intracellular mitochondrial ROS produced in these cells ultimately promotes extracellular oxidative stress during MS needs additional clarification. Furthermore, peripheral blood mononuclear cells during MS can upregulate 12/15-lipoxygenase^58^, an enzyme that directly oxidizes PCs^59^. These findings suggest that monocytic cells have the capacity to promote both enzymatic and non-enzymatic mediated OxPC formation. Consistent with this hypothesis, the greatest accumulation of monocyte derived macrophages with proinflammatory and pro-oxidative phenotypes in GM lesions coincided with the peak of neuronal loss at day 3. Conversely, microglia only became more abundant than monocyte derived macrophages in the GM lesion after day 7. More importantly, microglia depletion using the CSF1R inhibitor PLX3397 exacerbated GM lesion pathology, indicating that they helped to protect the GM against oxidative injury. Indeed, in middle-aged mice which have impaired microglial response compared to young mice, neurodegeneration and OxPC accumulation in GM lesions were more severe. Collectively, these findings are consistent with previous reports in the CNS WM showing that inflammatory monocytes and monocyte derived macrophages propagate early neuroinflammation during acute CNS injury whereas tissue resident microglia are more essential for repair, debris clearance, and neuroprotection^22,23,60^.

Iron deposition associates with progressive MS pathology^19,50,51^ and iron(ii) reacting with PCs through the Fenton reaction^52^ is another potential mechanism to generate OxPCs in the CNS. Aberrant iron accumulation in MS may be caused by oligodendrocyte cell death^18^ and iron loaded microglia/macrophages^50^, as well as by cerebral microbleeds^53^ and dysfunctional erythrocytes^54,55^, which can deposit iron-containing hemoglobin in the CNS. In human plasma, heme concentration can range from approximately 22 to 1056 μM^61^ which equates to approximately 13.5 to 651 μg/mL. Acute neurodegeneration induced by the injection of 5 μg of iron(ii) containing heme in the spinal GM could represent between 7 to 370 μL of blood plasma, which is likely within the physiological range since cerebral microbleeds detected by magnetic resonance imaging are typically 2 to 10 mm in diameter^62^. Thus, we provide proof-of-principle evidence that extracellular deposition of iron(ii) containing heme within the physiological range may be a plausible mechanism that promotes endogenous OxPC formation and drives neurodegeneration in the CNS GM. Given broad-rim lesions enriched with iron and oxidative innate immune response can help to predict the rate of MS progression^50^, in-depth histopathological characterization of iron deposition in the GM may provide further insights into the pathophysiology of GM neurodegeneration during progressive MS.

While stereotaxic deposition of MS-relevant OxPCs, such as POVPC, into the spinal cord GM is a useful model to recapitulate oxidative injury in MS, the resulting acute lesion likely best represents active GM pathology associated with significant oxidative inflammation and therefore may be less informative on what initiates new injury in the otherwise healthy GM during MS. Furthermore, quantitative analyses estimate that OxPC levels in MS range from ~1.5 nmol/g (~1.5 μM) of protein as measured by liquid chromatography–mass spectrometry (LC/MS)^26^ to ~1 μmol/g (~1000 μM) of protein as measured by immunoblotting^63^. This variable range is potentially because LC/MS preferentially detects individual molecular species of OxPCs^64^ whereas immunoblotting using OxPC specific antibodies such as E06 IgM captures OxPCs as well as OxPC adducts to peptides or proteins^36^. In this study, ~8.4 nmol of POVPC was injected into the mouse spinal cord to model GM neurodegeneration and additional comparative studies are needed to determine whether the resulting tissue concentration fully recapitulates OxPC levels and OxPC associated pathology found within MS lesion microenvironments. But given OxPCs induce cytotoxicity in different types of cells at concentrations ranging from 0.1 μM to 50 μM^22,35,44,65^, tissue exposure is likely detrimental to cellular homeostasis even at sub-μM concentrations, especially in an inflammatory microenvironment. Thus, additional investigation into how factors such as OxPC concentration, duration of exposure, or cytokine signaling affect the sensitivity of neurons and glial cells to OxPCs will be critical for understanding why the CNS may become more vulnerable to oxidative stress with age or during MS.

In summary, OxPC deposition in the CNS GM promotes endogenous lipid peroxidation, neuroinflammation, and RIPK3 dependent neuronal death. Iron(ii)-containing heme induced similar pathology in the GM, providing evidence for a potential link between iron deposition, OxPC formation, and neurodegeneration in MS. Aging and loss of microglia also exacerbated OxPC induced GM neurodegeneration, further demonstrating that they are risk factors for MS progression. These findings suggest GM neurodegeneration in MS is modulated by multiple factors and thus a combination of therapeutic strategies including iron chelation, OxPC neutralization, necroptosis inhibition, and microglial rejuvenation may be required to effectively stop MS progression especially in older individuals.

## METHODS

### MS Specimens

Post-mortem human brain tissues were obtained from six patients (aged 48–65 years old, 2 females and 4 males) diagnosed with clinical and neuropathological secondary progressive MS according to the revised 2010 McDonald’s criteria^66,67^, with full ethical approval (BH07.001, Nagano 20.332 - YP) and informed consent as approved by the CRCHUM and University of Montreal research ethics committee. As previously described^46,47^, autopsy samples were cryopreserved, and lesions were classified using Luxol fast blue and H&E staining.

### Mice

All experiments were conducted with ethics approval (protocol number 20220103) from the Animal Care Committee at the University of Saskatchewan under regulations of the Canadian Council of Animal Care. Six-week-old Female C57Bl/6NCrl mice were acquired from Charles River Laboratories (strain 027) and were used for most in vivo experiments between 8–12wk old, whereas female 6wk and 52wk old C57Bl/6J mice were acquired from the Jackson Laboratory (strain 000664) for experiments studying the effect of age. Tmem119^creERT2^ mice (strain 031820)^68^, Ai9^TdTom^ mice (strain 007909)^69^, and Rosa26^iDTR^ mice (strain 007900)^70^ from the Jackson Laboratory were bred in the Lab Animal Services Unit at the University of Saskatchewan to produce male and female Tmem119^CreERT2^:Ai9^TdTom^ mice and Tmem119^CreERT2^:Rosa26^iDTR^ mice for microglial lineage tracing and depletion studies, respectively. Mice were maintained on a regular diet in a low humidity environment on a 12-hr light/dark cycle at 21 to 23 °C with unlimited access to food and water. Mice and littermates were randomly assigned to different experimental groups.

### Spinal cord surgery

OxPC injection into the spinal cord GM was performed as described previously^22,71^, except that the depth of the injection was modified to target the GM rather than then WM. In brief, mice were anesthetized with subcutaneous injection of ketamine (100 mg/kg) and xylazine (10 mg/kg). Metacam (5 mg/kg) was injected subcutaneously as analgesic. Following dissection to expose the spinal cord between the T3 and T4 vertebrae, 0.5 μL of 10 mg/mL of POVPC (Sigma, 870606P) diluted in sterile PBS was injected at a depth of 0.9 mm with a rate of 0.25 μL/min. After injection, the needle was retained in place for 2 minutes to prevent liquid backflow. The mouse was then sutured, and bupivacaine (5 mg/kg) was applied along the suture as additional analgesic. Mice were also injected with atipamezole (100 mg/kg) intraperitoneally to reverse the effect of xylazine as well as slow-release buprenorphine (1 mg/kg) subcutaneously for extended pain relief. Finally, mice were placed in a thermally controlled environment for recovery.

### Iron(ii)-containing heme preparation

In a biosafety cabinet and avoiding light, iron(ii)-containing heme or ferroprotoporphyrin (Cayman Chemicals 34327) was first dissolved in 0.1M NaOH and then diluted 1:10 in sterile PBS (pH 7.4) to a final concentration of 2 mg/mL or 10 mg/mL. Solutions were then sterile filtered with a 0.22 μm syringe filter (Fisher Scientific) immediately prior to use. Using the spinal cord surgery method described above to induce spinal cord GM lesions, 0.5 μL of 2 mg/mL solution was injected into the spinal cord GM for a final dose of 1 μg per mouse whereas 0.5 μL of 10 mg/mL solution was injected for a final dose of 5 μg per mouse.

### Lineage tracing microglia

Six-week-old Tmem119^CreER^:Ai9^tdTom^ mice were intraperitoneally injected with 2 mg of tamoxifen (20 mg/mL; T5648, Sigma) dissolved in corn oil (C8267) once a day for 5 consecutive days followed by another 5 consecutive days with 2 days in between two set of injections to induce Tdtomato expression in Tmem119^+^ microglia. Tamoxifen treated mice were used for experiments 3 days after the final tamoxifen injection.

### Microglia depletion

Tmem119^CreERT2^: Rosa26^iDTR^ mice were injected intraperitoneally with 2 mg tamoxifen (20 mg/ml; T5648, Sigma) dissolved in corn oil (C8267) once a day for 5 consecutive days to induce diphtheria toxin receptor expression in microglia. Three days after the final injection, tamoxifen treated Tmem119^CreERT2^: Rosa26^iDTR^ mice were injected intraperitoneally with either PBS as controls or 1 μg of diphtheria toxin daily from days 0 to 7 after POVPC spinal cord injection.

Alternatively, C57BL/6NCrl mice were given normal chow or chow (Envigo) formulated with 300 mg/kg the CSF1R inhibitor PLX3397^40^ (Adooq) ad libitum for 2 weeks prior to experiments. After POVPC injection in the spinal cord GM, mice were kept on control or PLX3397 diet for another 7 days until tissues were collected for analysis.

### GSK-872 inhibition of RIPK3

GSK-872 (MedChemExpress HY-101872) was dissolved in DMSO into a 10 mM stock solution in DMSO for storage. For experiments, the stock solution was diluted with corn oil so that the final working solution is 10% DMSO and 90% corn oil. Mice were intraperitoneal injected with 20 mg/kg of GSK-872 once a day after POVPC injection and tissues were collected after 3 days for analysis. Mice treated with 10% DMSO and 90% corn oil without GSK-872 were used as controls.

### Spinal cord tissue isolation for histology and microscopy analysis

One-, 3-, 7-, or 14-days post spinal cord injection, mice were overdosed with isoflurane. Thereafter, 10 mL of ice-cold PBS followed by 10 mL of ice-cold 4% paraformaldehyde in PBS were perfused via cardiac puncture. The spinal cord was then dissected from the back of the mouse, and the tissue containing the lesion was collected into 4% paraformaldehyde in PBS for fixation overnight at 4 degrees. Afterwards, spinal cords were transferred to 30% sucrose solution for dehydration for at least 48 h and frozen in FSC 22 Frozen Section Media (Leica). Using a cryostat (ThermoFisher Scientific), spinal cord tissue was then cut coronally into 20 μm sections and collected on to Superfrost Plus microscope slides (VWR). All tissues were stored at −20 degrees prior to staining and analysis.

### Antibodies

The following primary antibodies were used for immunofluorescence microscopy: mouse E06 IgM anti-OxPC (1:200, Absolute Antibody Ab02746-21.0 and Sigma MABS2278), chicken anti-human/mouse IBA1 (1:500, Synaptic Systems 234009), rabbit anti-mouse IBA1 (1:1000, Wako 019-19741), chicken anti-mouse GFAP (1:1000, Biolegend 829401), rabbit anti-mouse p22phox (1:200, Cell Signaling 37570S), rat anti-human/mouse NeuN (1:400, Abcam ab177487), rabbit anti-mouse cleaved caspase-3 (1:200, Cell Signaling 9661S), rabbit anti-mouse pRIPK3 (1:300, Cell Signaling 91702S), rabbit anti-mouse pMLKL (1:500, Cell Signaling 37333T), rabbit anti-human pMLKL (1:100, R&D Systems MAB9187), rat anti-mouse CD11b (1:200, ThermoFisher 14-0112-82), rat anti-mouse iNOS (1:100, ThermoFisher 14-5920-82), rabbit anti-mouse Arg1 (1:200, Cell Signaling 93668S), rabbit anti-human/mouse Ki67 (1:200, Abcam ab15580), rabbit anti-mouse GluA2 (1:500, Abcam ab133477), rabbit anti-mouse VGLUT2 (1:500, Synaptic Systems 135408), mouse anti-mouse Tuj1 (1:400, Biolegend 801202), rabbit anti-mouse TNF-α (0.34 μg/mL, Cell Signaling 11948), rabbit IgG isotype control (0.34 μg/mL, ThermoFisher 02–6102).

The following secondary antibodies from Jackson ImmunoResearch were used at 1:400 dilution: Alexa Fluor 488 donkey anti-mouse IgM, Alexa Fluor 647 donkey anti-mouse IgM, Alexa Fluor 488 donkey anti-mouse IgG, Cyanine Cy3 donkey anti-chicken IgY, Alexa Fluor 647 donkey anti-rat IgG, Cyanine Cy3 donkey anti-rat IgG, Alexa Fluor 488 donkey anti-rabbit IgG, and Alexa Fluor 647 donkey anti-rabbit IgG.

### Labeling tissues for immunofluorescence microscopy

As previously described^22–25^, slides with mouse spinal cord samples were warmed to room temperature (RT) for 30 min. Tissues were rehydrated with PBS for 10 min and permeabilized with PBS with 0.2% Triton X-100 (Sigma) for 10 min. Blocking buffer (PBS, 10% donkey serum, 0.1% cold water fish gelatin, 0.1% Triton X-100, 0.05% Tween-20) was used to block non-specific binding for 1 h at room temperature in a humidity chamber. Alternatively, E06 IgM antibody staining, 5 μg/ml of purified Rat Anti-Mouse CD16/CD32 Fc blocking antibody (5 μg/ml, BD Pharmingen) was added to the blocking buffer. Afterwards, primary antibodies diluted in antibody dilution buffer (PBS, 0.1% cold fish gelatin, and 0.5% Triton X-100) were added onto microscopy slides and incubated overnight at 4 degrees. Next, slides were washed using PBS with 0.05% Tween-20 (Sigma) thrice for 5 minutes. Thereafter, tissues were incubated with secondary antibodies and DAPI (2 μg/mL, Sigma) diluted in antibody dilution buffer for 1 h at room temperature. For samples with potential high autofluorescence, slides were also blocked using the TrueBlack Lipofuscin Autofluorescence Quencher (Biotium) in accordance with manufacturer’s instructions. Finally, slides were washed thrice using PBS with 0.5% Tween-20, 5 min each, and coverslips were mounted onto the slides using Fluoromount-G solution (SouthernBiotech).

### Immunofluorescence microscopy

Immunofluorescence images were acquired at room temperature using the Zeiss Axio Observer 7 widefield microscope with Colibri 7 LED illumination and a 10 × 0.45NA air objective for all samples, followed by default background subtraction. Excitation wavelengths at 405 nm, 488 nm, 552 nm and 640 nm were used to excite fluorophore-conjugated on secondary antibodies. Image acquisition settings including pixel size (0.274 μm/pixel), bit rate, binning (1×1), exposure time, and gain (4X) were kept equal across all samples for each experiment to prevent sample collection bias. A sample slide stained with only secondary antibodies and DAPI was used for each experiment to control for non-specific secondary immunofluorescence. The lesion epicenter as determined by the largest lesion area in any given section for each mouse was acquired for data analysis. Alternatively, images of all serial sections that contain lesions (each 400 μm apart) for each spinal cord were acquired for lesion volume analysis. Zeiss Zen (v3.9) software was used for image acquisition.

### Image analysis

Images were analyzed using ImageJ 1.54f (Fiji, NIH) as previously described^25^. In brief, the raw grey scale images for each channel/marker were converted to RGB, and region of interest (ROI) was drawn around the mouse spinal cord grey matter on the ipsilateral GM side where POVPC was injected (also contralateral NAGM for certain analyses). To analyze immunoreactivity within GM, the area outside the ROI was first cleared, and the same color threshold values were used consistently to identify positive signals for each channel/marker across all samples in each set of experiments to prevent analysis bias. The analyze particles function was then used to quantify the positive signals in each ROI. The same size and circularity settings for particle analysis were used for all samples in each experimental set. For representative images shown, channels/markers for each sample were merged and displayed using pseudo colors. Only brightness and contrast settings were adjusted in ImageJ, and consistently between samples for better displaying the images.

### Mouse cortical neuron culture

Mouse-derived neurons were cultured as previously described^72^. Briefly, brains were collected from mice embryos at embryonic days E16–18, and cortical neurons were isolated and cultured in neurobasal plus medium (ThermoFisher, 21103049) with B-27 plus supplement (ThermoFisher, A3653401) in T-25 tissue culture flasks (BD Falcon). After 2 days in culture, cortical neurons were detached from the T-25 flasks using 1x Versene solution with 0.25% trypsin and 1 mg/mL DNase I and seeded onto 96-well plates at a density of 5 × 10^4^ neurons per well, and plates were incubated at 37 degrees in a 5% CO_2_ incubator. Five to 6 days later, neurons were treated with either media alone as the control, with 50 μM PC (Sigma, P1652), or 50 M POVPC for 24 h. Afterwards, neurons were fixed with PBS containing 4% paraformaldehyde for 30 minutes at room temperature and were washed with PBS thrice for 5 min. For immunofluorescence microscopy analysis, cells were permeabilized in 1 × PBS with 0.2% Triton-X for 10 minutes, then incubated in donkey blocking buffer for 1 h before incubating in primary antibodies overnight in 4 degrees. Thereafter, cells were washed in 1 × PBS with 0.05% Tween-20 thrice for 5 minutes and incubated with antibody dilution buffer containing secondary antibodies and DAPI for 1h at room temperature. Finally, cells were washed in PBS with 0.05% Tween-20 thrice for 5 min and resuspended in PBS. Images of labeled neurons in 96 well plates were acquired using the Zeiss Axio Observer 7 widefield microscope as described above. Specifically, 9 fields-of-views were collected per well for quantitative analysis as described above.

### Statistics and reproducibility

All data were collated in Microsoft Excel (v2605) and graphs were generated using GraphPad Prism 10.6.1 (LaJolla, CA). Data are shown as individual data points where each point on a graph represents a separate mouse for *in vivo* experiments, or a separate well for neuron culture experiments. The mean ± SD are also shown. While no power calculation was performed, the sample size was determined based on previously published results^22–25^ as well as the cost of experiment, feasibility of the experiment, as well as the availability of sex and aged matched mice. Approximately 5 percent of mice in experiments had failed injections with no apparent GM injury, and these animals were not analyzed. Otherwise, no data was excluded from the study. Sample sizes are reported in the figure legends and only one measurement is recorded per sample. Littermate mice were selected randomly for each experiment and treatment groups. Data collection and analysis were not performed blindly to the experimental condition since quantitative analyses were performed using equal acquisition and analysis settings across all samples for each experiment. To analyze the statistical differences between the means of two or more treatment groups against each other, one-way ANOVA with Tukey’s multiple comparison test was used, while one-way ANOVA with Dunnett’s multiple comparisons test was used to compare two or more treatment groups against the control group. Two-tailed, unpaired t-test was used to compare data with only two groups. Kolmogorov-Smirnov test was used to verify the normal distribution of data. Specific p-values are reported in each figure where results are considered statistically significant where p< 0.05.

## Extended Data

## Data Availability

Numerical raw data used for statistical analysis in all experiments from this study are available as supplementary material. All other data are available upon request.
